# Genome-Wide Association Study Reveals the Genetic Architecture of Growth and Meat Production Traits in a Chicken F_2_ Resource Population

**DOI:** 10.3390/genes15101246

**Published:** 2024-09-25

**Authors:** Natalia A. Volkova, Michael N. Romanov, Anastasia N. Vetokh, Polina V. Larionova, Ludmila A. Volkova, Alexandra S. Abdelmanova, Alexander A. Sermyagin, Darren K. Griffin, Natalia A. Zinovieva

**Affiliations:** 1 L. K. Ernst Federal Research Center for Animal Husbandry, Dubrovitsy, Podolsk 142132, Moscow Oblast, Russia; natavolkova@inbox.ru (N.A.V.); anastezuya@mail.ru (A.N.V.); volpolina@mail.ru (P.V.L.); ludavolkova@inbox.ru (L.A.V.); abdelmanova@vij.ru (A.S.A.); 2 School of Biosciences, University of Kent, Canterbury CT2 7NJ, UK; d.k.griffin@kent.ac.uk; 3 Animal Genomics and Bioresource Research Unit (AGB Research Unit), Faculty of Science, Kasetsart University, Bangkok 10900, Thailand; 4 Russian Research Institute of Farm Animal Genetics and Breeding—Branch of the L. K. Ernst Federal Research Centre for Animal Husbandry, Pushkin, St. Petersburg 196601, Russia; alex_sermyagin85@mail.ru

**Keywords:** chicken, GWAS, SNPs, candidate genes, growth, body weight, meat performance

## Abstract

Background/Objectives: For genomic selection to enhance the efficiency of broiler production, finding SNPs and candidate genes that define the manifestation of main selected traits is essential. We conducted a genome-wide association study (GWAS) for growth and meat productivity traits of roosters from a chicken F_2_ resource population (*n* = 152). Methods: The population was obtained by crossing two breeds with contrasting phenotypes for performance indicators, i.e., Russian White (slow-growing) and Cornish White (fast-growing). The birds were genotyped using the Illumina Chicken 60K SNP iSelect BeadChip. After LD filtering of the data, 54,188 SNPs were employed for the GWAS analysis that allowed us to reveal significant specific associations for phenotypic traits of interest and economic importance. Results: At the threshold value of *p* < 9.2 × 10^−7^, 83 SNPs associated with body weight at the age of 28, 42, and 63 days were identified, as well as 171 SNPs associated with meat qualities (average daily gain, slaughter yield, and dressed carcass weight and its components). Moreover, 34 SNPs were associated with a group of three or more traits, including 15 SNPs significant for a group of growth traits and 5 SNPs for a group of meat productivity indicators. Relevant to these detected SNPs, nine prioritized candidate genes associated with the studied traits were revealed, including *WNT2*, *DEPTOR*, *PPA2*, *UNC80*, *DDX51*, *PAPPA*, *SSC4D*, *PTPRU*, and *TLK2*. Conclusions: The found SNPs and candidate genes can serve as genetic markers for growth and meat performance characteristics in chicken breeding in order to achieve genetic improvement in broiler production.

## 1. Introduction

Over recent decades, there has been a trend towards an increase in the production and consumption of poultry meat relative to other meat products, with health, low fat, high protein, and a high concentration of polyunsaturated fatty acids typically cited [1,2]. Growth and production traits are hugely important to the poultry industry, with meat quality depending on a number of genetically determined factors [3,4,5]. Commercial crossbred broiler chickens are earlier in maturing and are characterized by a higher percentage of breast muscle compared to purebred chickens, especially local breeds [6,7,8]. At the same time, the meat of broilers and meat-type breeds may contain a greater amount of subcutaneous and abdominal fat compared to meat obtained from slow-growing breed chickens [9,10]. In order to maximize the potential of poultry breeding, a deeper understanding of the genetic factors that control growth and meat quality [11,12,13], and how they interact with environmental conditions involving rearing, keeping [14,15], and feeding [16,17] is essential. In other words, research progress on individual traits influencing environmental factors and the genetic mechanisms that govern them is of great value to the poultry industry and its worldwide consumers [18,19]. For commercial production of chicken meat [20], highly productive broiler crosses that are characterized by a high growth rate and good meat qualities are usually used [21,22]. As a result of extensive functional genomic research, birds of this type are now distinguished by effective feed conversion and high slaughter yield (SY) of both the carcass and its individual components, including breast weight (BrW) [23,24,25]. Directed selection for body weight (BW) contributed to an increase in the efficiency of meat production [26] by reducing the time it takes to grow birds while increasing the marketable weight and meat yield, including pectoral muscle weight [23,27].

A number of studies have demonstrated high heritability of growth traits in early-age broiler chickens [28,29,30]. Phenotypic selection for these traits can contribute to significant progress in broiler breeding and the creation of highly productive commercial crosses. A correlation between BW and carcass characteristics has been shown in commercial broiler lines [31]. Along with traditional selection methods, studies aimed at finding and identifying genetic markers associated with growth and other performance indicators in chickens are in demand [32,33]. Research in this area is crucial for understanding the genetic basis of growth traits in broiler chickens toward the subsequent implementation of effective breeding programs aimed at increasing genetic potential of commercial poultry. To date, significant progress has been made in the genetic study of indicators characterizing the growth rate, meat qualities, and other phenotypic traits of chickens [34,35,36].

With the development of high-density single nucleotide polymorphism (SNP) arrays, genome-wide association studies (GWAS) have been instrumental in identifying hitherto undiscovered genetic associations of SNPs with phenotypic traits in livestock [37,38,39]. This approach was broadly applied to seeking associations (especially with BW and BrW) and, thereafter, identifying related candidate genes [40,41,42]. In our earlier study [43], we analyzed potential genes and selective signatures in grandparent lines undergoing strong selection pressure for broiler productivity.

The purpose of this study was to extend this prior work to focus on the search for, and identification of, SNPs associated with growth and meat productivity parameters in chickens, such as BW, average daily BW gain (ADBWG), SY, dressed carcass weight (DCW), and weight of its components, including BrW and weights of thighs (TW), drumsticks (DW), and wings (WW). Of special interest was the search for significant SNPs and prime candidate genes common to several traits taken into account. In accordance with this goal, the GWAS analysis for growth parameters and meat qualities in roosters of a chicken F_2_ resource population was carried out based on genome-wide genotyping data. The F_2_ resource population was obtained by interbreeding the meat-type Cornish White (CW) breed characterized by fast growth [44] and the egg-layer Russian White (RW) breed of slow growth [45,46].

## 2. Materials and Methods

### 2.1. Birds Involved in the Experiment

Chickens of the original breeds were hatched from eggs obtained from Genofond LLC (All-Russian Poultry Research and Technological Institute, Sergiev Posad, Russia) and the Russian Research Institute of Farm Animal Genetics and Breeding (Pushkin, Russia), raised at the L. K. Ernst Federal Research Centre for Animal Husbandry (LKEFRCAH), and sampled for DNA. The F_2_ chickens of the resource population were produced and reared at the LKEFRCAH.

To obtain the F_2_ resource population, two breeds with contrasting growth rates and meat qualities were used: RW, of slow growth [47,48,49], and CW, of fast growth [43,44,50]. At the first stage, based on the data of genome-wide genotyping (to exclude close relationships), two families (F0_1 and F0_2) were formed from individuals of the original parental breeds, each of which contained one RW rooster and five CW females. Through interbreed crosses, F_1_ hybrids (*n* = 36) were produced from each family and chosen for further research. These interbred F_1_ hybrids were used to obtain F_2_ individuals. For this purpose, nine families, F1_1 to F1_9, were established, each of which included one F_1_ male and three F_1_ females that were not close relatives. The resultant F_2_ offspring (*n* = 152, males of groups F2_1 to F2_9) were utilized as a model resource population for further molecular genetic studies to search for SNPs associated with growth and meat productivity indicators of chickens.

F_2_ chickens were raised in brooders up to 3 weeks of age with a gradual temperature decrease from 34 °C (in the first hours post hatch) to 23 °C and then transferred to floor maintenance. Keeping the birds according to their age implied permanent access to complete commercial compound feed and fresh water, good supply ventilation (ensuring the absence of dampness, drafts, and gas pollution), and normal lighting [51,52].

### 2.2. Phenotypic Characteristics

F_2_ males of the resource population were phenotyped for the following growth and meat productivity parameters (in g): BW at the age of 14 (BW14), 28 (BW28), 42 (BW42), and 63 (BW63) days, ADBWG, SY, DCW, BrW, TW, DW, and WW. ADBWG was calculated for the growing period from 1 to 63 days. At the age of 63 days, the birds were experimentally slaughtered to evaluate the weight parameters of the carcass and its components using a laboratory scale. The carcass was cut into parts for further determining DCW, BrW, TW, DW, and WW. When measuring such traits as TW, DW, and WW, the mean value of these indicators established for each of the two thighs, drumsticks, or wings was calculated.

### 2.3. Sampling and DNA Extraction

Feather pulp was used to extract DNA. DNA isolation was executed using the DNA Extran kit for DNA isolation from animal tissues (Syntol, Moscow, Russia). The concentration of DNA solutions was determined using a Qubit 3.0 Fluorimeter (Thermo Fisher Scientific, Wilmington, DE, USA). The OD260/280 ratio was measured using the NanoDrop-2000 device (Thermo Fisher Scientific) to verify the isolated DNA’s purity.

### 2.4. SNP Genotyping and Quality Control

Whole-genome genotyping of chickens was performed using the Illumina Chicken 60K SNP iSelect BeadChip (Illumina, San Diego, CA, USA) containing 60 thousand SNPs. Quality control and filtering of genotyping data for each sample and each SNP were performed in the R-4.0 software environment [53] using the PLINK 1.9 software package [54,55], applying the following filters in the program: –mind 0.10, –geno 0.10, –maf 0.01, –hwe 1e-6. After pruning, 54,188 SNPs were retained for further analysis.

### 2.5. Principal Component Analysis

Principal component analysis (PCA; [56]) was performed and visualized in the R package ggplot2 [57,58]. Data files were prepared in the R-4.0 software environment [59].

### 2.6. GWAS Analysis

To identify SNP associations with growth and meat productivity indicators in the F_2_ resource population chickens, the respective regression analysis in PLINK 1.9 was used. Significance of the SNP effects and the identification of significant regions in the chicken genome were assessed using the Bonferroni null hypothesis test at a threshold of *p* < 9.2 × 10^−7^. The data were visualized in the qqman package (version 0.1.9) [60] using the R-4.0 programming language [61].

Search for candidate genes localized in the region of the identified SNPs (including 0.2-Mb flanks on both sides) was performed according to the chicken (*Gallus gallus*; GGA) reference genome assembly GRCg6a [62] and using the Genome Data Viewer in the NCBI chicken databases [63]. The web-based Ensembl Genes release 106 database and Ensembl BioMart data mining tool [64] were utilized to get detailed information for SNPs located within or near the candidate genes identified. To perform functional annotation and gene ontology (GO) term enrichment analysis for prime candidate genes, the Ensembl BioMart data mining tool and Database for Annotation, Visualization, and Integrated Discovery (DAVID Knowledgebase; version DAVID 2021 (December 2021), v2023q4, updated quarterly) [65,66] were exploited.

## 3. Results

### 3.1. Population Stratification

**Figure 1:**
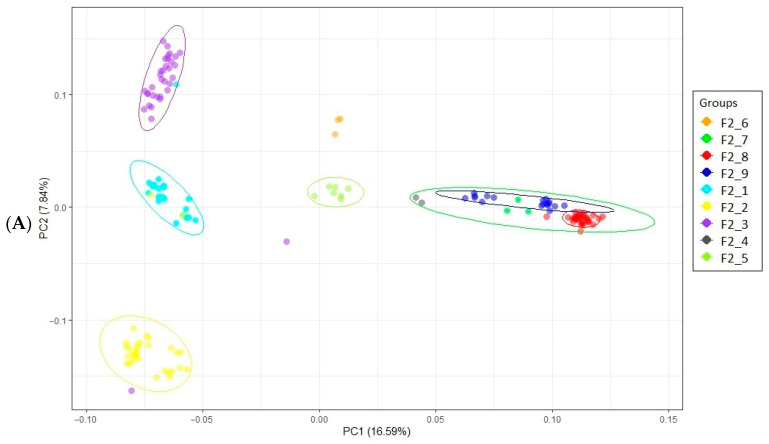

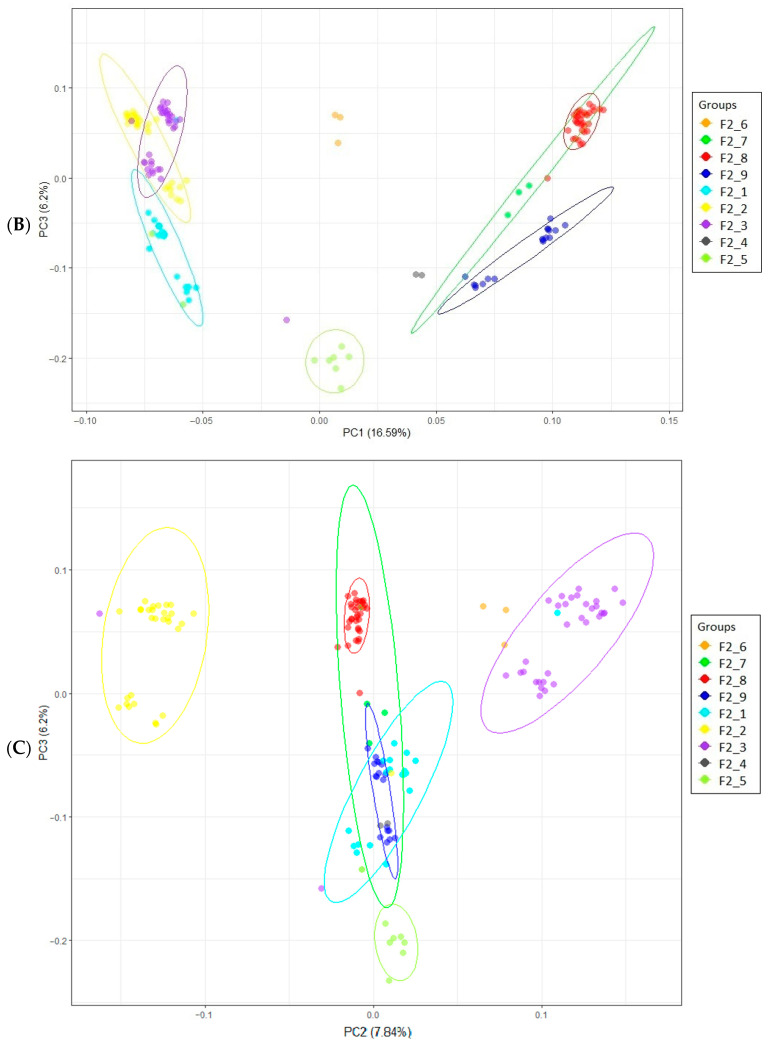

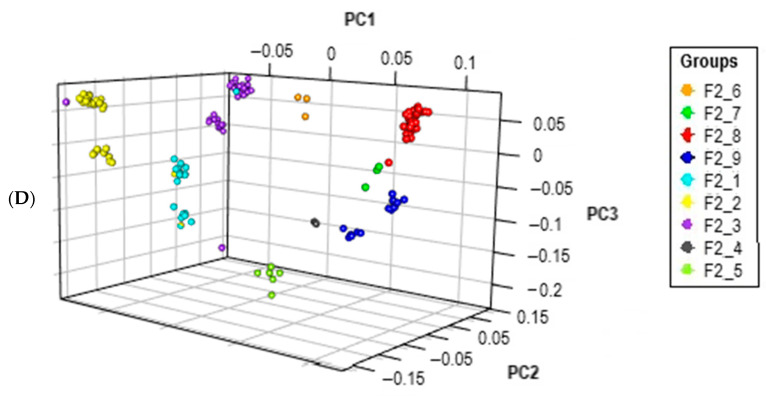
Principal component analysis for the chicken F_2_ resource population: (**A**) in the plane of the first (PC1; *X*-axis) and second (PC2; *Y*-axis) components; and (**B**) in the plane of the first (PC1; *X*-axis) and third (PC3; *Y*-axis) components; (**C**) in the plane of the second (PC2; *X*-axis) and third (PC3; *Y*-axis) components; (**D**); in a 3D chart with three components (PC1–PC2–PC3). Individuals from different groups are indicated by different colors.

PCA showed the distribution of the studied F_2_ resource population into several clusters. The first component (PC1) accounted for 16.57% of the genetic variability, the second component (PC3) for 7.84%, and the third component (PC3) for 6.20%. In the PC1–PC2 projection, the population under study was differentiated into five groups: the first group included F2_7, F2_9, F2_8, and F2_4 progenies, the second group F2_5, the third group F2_3, the fourth group F2_1, and the fifth group F2_2. In the PC1–PC3 projection, three groups were distinguished: the first group consisted of F2_3, F2_2, and F2_1 progenies, the second group of F2_7, F2_8, and F2_9 progenies, and the third group was evenly spaced from the previous two and included F2_5. This information is visually presented in Figure 1.

Given the observed population stratification, i.e., its revealed structure, we performed the GWAS using the first three PCs as covariates.

### 3.2. GWAS Results

Table 1 summarizes the data on the studied growth and meat productivity indices in F_2_ males of the resource population. In particular, descriptive statistics are presented that characterize the distribution of values established for the measured characteristics. Herein, the coefficient of variation of the values of the studied traits varied from 5.8 to 26.1%.

**Figure 2:**
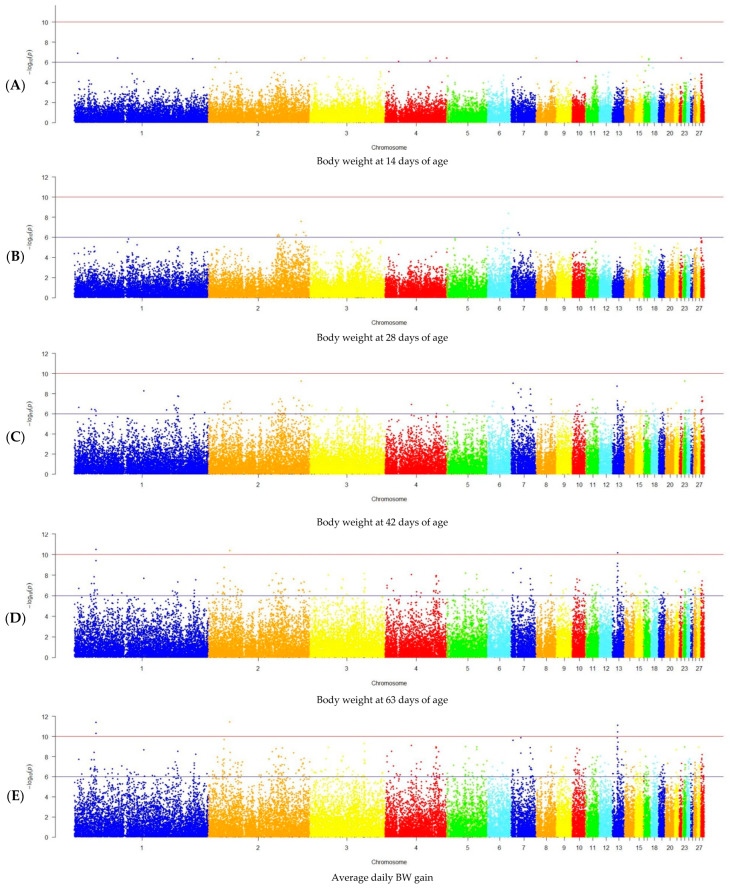

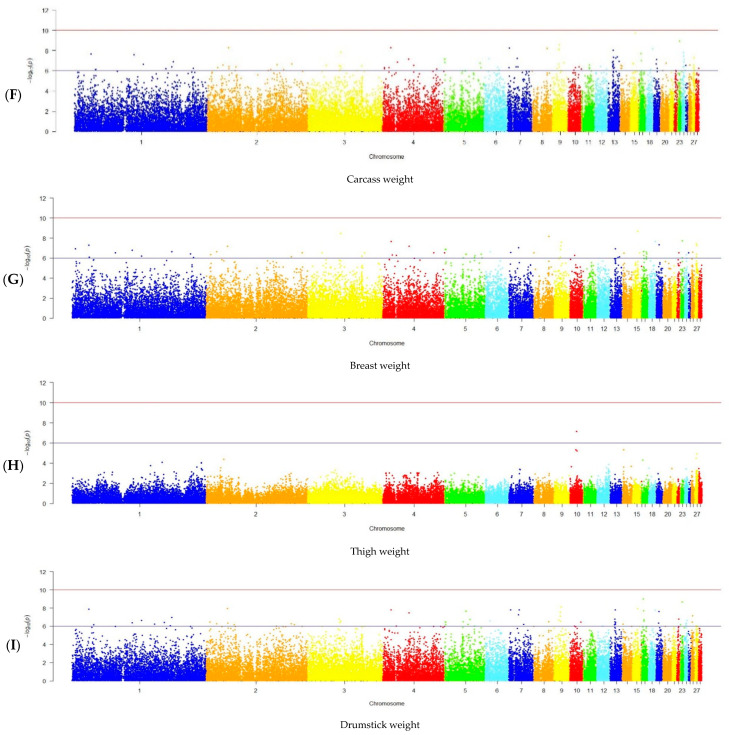
Manhattan plots for the studied growth and meat productivity parameters in the chicken F_2_ resource population: (**A**) body weight (BW) at 14 days of age, (**B**) BW at 28 days of age, (**C**) BW at 42 days of age, (**D**) BW at 63 days of age, (**E**) average daily BW gain, (**F**) carcass weight, (**G**) breast weight, (**H**) thigh weight, and (**I**) drumstick weight. Manhattan plots show the distribution of single nucleotide mutations in chicken chromosomes to the significance level (−log10 (*p*)) according to the expected probability value of *p* < 1.05 × 10^−6^ (blue line) and *p* < 1.05 × 10^−10^ (red line) for the studied traits. Dots are color-coded only to visualize chromosome segregation.

The obtained phenotypic data for growth and meat productivity in F_2_ males of the resource population were used for the GWAS. The GWAS results are presented in Figure 2.

The conducted analysis revealed 83 SNPs associated with the BW of chickens in the studied population at the age of 28, 42, and 63 days and 171 SNPs associated with the meat productivity parameters at the threshold level of the established significance value *p* < 9.2 × 10^−7^ (Supplementary Table S1). These SNPs were observed on 27 chromosomes. Herein, the maximum number of identified SNPs was localized on chromosomes GGA1, GGA2, and GGA13 (18, 37, and 15 SNPs, respectively), while the minimum SNP number (1–2 SNPs) on GGA8, GGA14, GGA15, GGA17, GGA19–GGA23, GGA25, and GGA27. On GGA16, no significant SNPs were found for any of the examined parameters. Data on the number of identified significant SNPs and their distribution on chromosomes, taking into account each specifically growth and meat productivity indicator studied in the chicken F_2_ resource population, are presented in Table 2.

The GWAS for BW parameters in F_2_ males of the studied population returned the result of 2, 34, and 69 SNPs associated with this trait at the age of 28, 42, and 63 days, respectively. The maximum number of SNPs was established on GGA1 and GGA2 (11 and 20, respectively), and the minimum (1 SNP) on GGA6, GGA8, GGA14, GGA15, GGA17, GGA21, GGA23, and GGA27. Analysis for ADBWG in the period from 1 to 63 days of age revealed 148 significant SNPs associated with this parameter. Similar to the GWAS results for the BW trait, the maximum number of these SNPs was detected on GGA1 and GGA2 (15 and 33 SNPs, respectively).

The number of significant SNPs associated with the examined weight parameters of the carcass and its components varied from 16 to 30, with the exception of TW for which only one SNP was determined on GGA10. The maximum number of SNPs localized in the specific chromosomes was found for the following traits: DCW on GGA13 (5 SNPs); BrW on GGA4, GGA7, GGA9, and GGA27 (2 SNPs); and DW on GGA7 (5 SNPs). The minimum SNP number (1–2 SNPs) was identified for the following traits: DCW on GGA2, GGA3, GGA6, GGA8, GGA15, GGA17–GGA19, GGA23, and GGA27; BrW on GGA1, GGA2, GGA3, GGA8, GGA15, GGA18, GGA19, and GGA23; and DW on GGA1, GGA2, GGA13, GGA15, GGA18, GGA19, GGA23, and GGA26. For the two studied parameters—SY and WW—no significant SNPs were observed at the established significance threshold.

Comparative analysis of the defined genomic associations with growth and meat productivity indicators in F_2_ roosters of the resource population demonstrated the presence of SNPs common to the group of traits assessed in this investigation (Table 2). In particular, 22 SNPs associated with any three traits were identified. Herein, we found 15 common SNPs significantly associated with growth indicators (BW42, BW63, and ADBWG) and five SNPs associated with meat qualities (DCW, BrW, and TW). The number of SNPs common to four, five, and six traits was five, three, and four SNPs, respectively. These SNPs were significantly associated with a group of traits including both growth indicators and meat productivity. For one of the traits studied in this study, TW, no SNPs were found in common with the other traits studied. For one of the traits investigated in this study, i.e., TW, no SNPs were detected in common with the other traits studied.

### 3.3. Candidate Genes

SNPs established jointly for a group of studied traits (3–6 traits) were used to annotate prime candidate genes associated with growth and meat productivity in broiler chickens. Structural annotation in the area of identified SNPs (i.e., SNP position ± 0.2 Mb) resulted in 239 genes described in the NCBI databases. These candidate genes are listed in Supplementary Table S1, with their locations indicated in the flanking regions relative to the respective SNPs or at exact SNP position. Supplementary Table S2 also shows that most genes overlapping the SNP positions contained polymorphic variants of these SNPs in introns, plus one gene with a synonymous (exonic) variant and one gene with a 5′ UTR variant. Herein, there were the following nine prioritized candidate genes (PCGs) localized at the positions of the SNPs identified for three and more traits: *WNT2* (Wnt family member 2), *DEPTOR* (DEP domain containing MTOR-interacting protein), *PPA2* (inorganic pyrophosphatase 2), *UNC80* (unc-80 homolog, NALCN activator), *DDX51* (DEAD-box helicase 51), *PAPPA* (pappalysin 1), *SSC4D* (scavenger receptor cysteine rich family member with 4 domains), *PTPRU* (protein tyrosine phosphatase, receptor type U), and *TLK2* (tousled-like kinase 2). These PCGs are located on the following nine chromosomes: GGA1, GGA2, GGA4, GGA7, GGA15, GGA17, GGA19, GGA23, and GGA27. Candidate genes, including PCGs, and significant SNPs (*p* < 9.2 × 10^−7^) associated with growth and meat productivity indicators in F_2_ roosters of the resource population are shown in Table 3.

Based on the GO term enrichment assessment, the annotated genes were grouped into six functional clusters. However, three clusters had Enrichment Scores below one, so we considered them insignificant. The other three clusters (with Enrichment Scores > 1.2) included genes associated with peptidyl-serine phosphorylation, kinase, and lipoprotein. The list of annotated genes and their functions are presented in Supplementary Table S2.

## 4. Discussion

Identification and mapping of genes determining the manifestation of economically important traits in poultry is one of the key tasks of genomic selection aimed at increasing the efficiency of poultry production [67,68,69]. The GWAS approach manifested in this study is pivotal in elucidating the genetic mechanisms determining BW and muscle production traits in chickens [70], thereby having the potential to increase the efficiency of poultry production [71,72,73,74,75]. Here, the resource population was obtained by interbreeding two breeds with contrasting productivity indicators, i.e., RW (slow-growing) and CW (fast-growing). Although these traits, to a certain extent, depend on the nature of the feed given to the animals as well as their housing conditions, they are also genetically determined by multiple QTLs [76]. We identified significant SNPs (*p* < 9.2 × 10^−7^) associated with growth and meat performance in F_2_ roosters of the resource population. In particular, BW28, BW42, and BW63 (2, 34, and 69 SNPs, respectively); ADBWG (148 SNPs); DCW (30 SNPs); BrW (16 SNPs); and DW (21 SNPs) were implicated, with the greatest number of identified SNPs localized on the largest two chromosomes (GGA1 and GGA2).

For practical use in genomic selection, it is essential to identify SNPs and prime candidate genes associated with a small number of selected traits [77,78,79]. Here, we identified such SNPs significant for specific growth and meat productivity indicators. In particular, associations with 15 SNPs characterizing growth (BW42, BW63, and ADBWG) were identified, alongside five SNPs for meat performance traits (DCW, BrW, and TW). Moreover, 14 SNPs were identified associated with traits characterizing *both* growth indicators and meat productivity.

Regarding the positions of the identified SNPs and likely causative alleles for three or more traits, nine PCGs were located, including *WNT2*, *DEPTOR*, *PPA2*, *UNC80*, *DDX51*, *PAPPA*, *SSC4D*, *PTPRU*, and *TLK2*. As shown in other investigations, of these nine, four (*UNC80*, *TLK2*, *PTPRU*, and *DDX51*) were associated with growth indicators in farm animals, including poultry. Three of these genes (*TLK2*, *PTPRU*, and *DDX51*) were associated with BW and BrW in chickens. These results tally with Zhang et al.’s [80], who explored growth indicators in Jinghai Yellow chickens at the age of 2, 4, 6, 8, 12, 14, and 16 weeks based on genomic data using the same Illumina Chicken 60K SNP array. In accordance with their GWAS analysis, a significant association of the *PTPRU* gene (*p* < 1.80 × 10^−6^) with BW of chickens at the age of 12 weeks was established. Walugembe et al. [81] investigated the growth indicators of chickens infected with lentogenic Newcastle disease virus. An association of the *DDX51* gene with the BW of chickens before infection was revealed. Kang et al. [82] established an association of the *TLK2* gene with BrW in broiler chickens at the age of 126 days. A number of other studies have shown associations of the *TLK2* gene with BW in cattle [83] and that of the *UNC80* gene with BW in sheep at the age of 180 days [84].

The effects of the genes *PPA2*, *SSC4D*, and *PAPPA* on the growth rates and meat productivity established in F_2_ roosters of the resource population in this study has also confirmed other studies. In particular, it was shown that there was an influence of the *PPA2* gene on feed consumption in quails [85] and pigs [86,87], that of the *SSC4D* gene on the hip height in cattle at the age of 18 and 24 months [88], and that of the *PAPPA* gene on the body length and depth in pigs [89]. Feed consumption and body measurements are associated with the growth rates in several animals, including poultry, as the feed consumption indicator affects the growth, while linear body measurements correlate with BW and body size.

Along with feed consumption and linear measurements, BW and meat qualities of animals, including poultry [90,91,92], are also associated with the fatty acid metabolism index that determines the fat content in the carcass as well as the taste of the meat [93,94,95]. A number of studies have demonstrated a relationship between the *PTPRU* gene and the abdominal fat weight in broilers [96], the content of flavor-presenting aldehydes related to the meat taste in chickens [97], and intramuscular fatty acid composition in pigs [98].

The growth and development of animals, including poultry, can be affected, to a certain extent, by immunity that governs resistance to infectious diseases, as well as adaptation to environmental conditions [37,71,81,99]. Of the prime candidate genes identified in the present study, some other investigations have shown a connection between the *PPA2* gene and the ability of sheep to adapt to high-altitude conditions [100] and a relationship of the *WNT2* and *TLK2* genes with resistance to infectious diseases in cattle [101] and chickens [81].

Thus, the available findings from other studies are largely concordant with the data we obtained on the direct effects of the genes *UNC80*, *TLK2*, *PTPRU*, and *DDX51* on the growth and meat productivity in chickens. For other PCGs identified in our work, a number of observations have also shown their connection with selected traits in other farm animals, including poultry. We also analyzed all genes overlapping significant SNP regions revealed in the GWAS for functional enrichment (Supplementary Table S2), based on the idea that genes interacting within similar biological networks may collaboratively influence the growth/meat performance phenotype [102]. GO analysis illustrated that prime candidates were enriched in relation to peptidyl−serine phosphorylation, kinase activity, and membrane lipoprotein component that have broad biological/metabolic roles [64,65,66]. Further research using GWAS and whole-genome sequencing approaches [102] is required to confirm the association of these PCGs with the growth and meat performance in chickens.

## 5. Conclusions

In this work, we performed a GWAS for parameters related to growth and meat productivity in F_2_ roosters of the resource population using the Illumina Chicken 60K SNP iSelect BeadChip. SNPs, and the respective prime candidate genes, showing significant association with BW at the age of 28, 42, and 63 days, and meat qualities of the studied birds at the age of 63 days were identified using the characterized genetic variants. The maximum number of identified SNPs was observed on GGA1, GGA2, and GGA13 (15–37 SNPs), while their minimum number was revealed on chromosomes GGA8, GGA14, GGA15, GGA17, GGA19–GGA23, GGA25, and GGA27 (1–2 SNPs). Herein, 34 SNPs were found that were common to three or more traits examined in this work. Nine PCGs that have biological functions potentially relevant for growth and meat performance were identified at these SNP positions: *WNT2*, *DEPTOR*, *PPA2*, *UNC80*, *DDX51*, *PAPPA*, *SSC4D*, *PTPRU*, and *TLK2*. These data are of great importance for understanding the genetic basis for the formation and manifestation of growth and meat qualities in chickens. The identified SNPs and PCGs warrant further investigation and can be used as genetic markers in breeding programs aimed at increasing growth rates and improving meat performance.

## Figures and Tables

**Table 1:** Descriptive statistics ^1^ for growth and meat performance indicators (in g) in F_2_ roosters of the resource population.

Trait	Mean	SD	Min–Max	CV, %
BW at 14-day age, g	215.7	45.7	92.8–396.1	21.2
BW at 28-day age, g	611.6	111.7	341.6–902.4	18.3
BW at 42-day age, g	1132.9	207.4	644.2–1690.1	18.3
BW at 63-day age, g	1829.1	377.9	963.9–2747.7	20.7
Average daily BW gain, g	28.7	6.1	14.6–43.0	21.4
Slaughter weight, %	71.1	4.1	55.4–80.1	5.8
Dressed carcass weight, g	1346.4	309.1	665.3–2032.1	23.0
Breast weight, g	385.3	100.5	144.8–632.6	26.1
Thigh weight, g	104.1	25.8	49.4–163.2	24.8
Drumstick weight, g	88.6	18.3	42.7–133.3	20.6
Wing weight, g	77.8	15.8	33.4–119.1	20.3

^1^ BW, body weight; SD, standard deviation; min, minimum; max, maximum; CV, coefficient of variation.

**Table 2:** Distribution of significant SNPs (*p* < 9.2 × 10^−7^) across chromosomes there were associated with body weight (BW) and meat productivity in the chicken F_2_ resource population.

Trait	No. of SNPs	Chromosomes
BW at 14-day age	-	-
BW at 28-day age	2	2, 6
BW at 42-day age	34	1, 2, 6–8, 11, 13–14, 21, 23, 28
BW at 63-day age	69	1–5, 7, 8, 10, 11, 13–15, 17, 21, 23, 27, 28
Average daily BW gain	148	1–15, 17, 18, 20–28
Slaughter weight	-	-
Dressed carcass weight	30	1–9, 13, 15, 17–19, 23, 24, 27
Breast weight	16	1-4, 7-9, 15, 18, 19, 23, 27
Thigh weight	1	10
Drumstick weight	21	1–2, 4–5, 7, 9, 13, 15, 17–19, 23, 26
Wing weight	-	-

**Table 3:** SNPs and prime candidate genes (*p* < 9.2 × 10^−7^) associated with growth and meat productivity in the chicken F_2_ resource population.

GGA ^1^	SNP	Position, bp	Traits ^2^	Genes
1	Gga_rs14800862	24,842,665	DCW, BrW, DW	*CTTNBP2*, *CFTR*, *ASZ1*, ***WNT2***, *ST7*, *CAPZA2*
1	Gga_rs14902811	152,430,990	BW42, BW63, ADBWG	-
1	Gga_rs14902833	152,488,231	BW42, BW63, ADBWG	*SLC2A13*
1	GGaluGA050529	152,453,938	BW42, BW63, ADBWG	*SLC2A13*
1	GGaluGA034658	102,412,092	BW42, BW63, ADBWG	-
2	Gga_rs14160005	31,441,781	BW42, BW63, ADBWG, DCW, BrW, DW	*IGF2BP3*, *TRA2A*, *CCDC126*, *FAM221A*, *STK31*, *NPY*, *PALS2*, *DFNA5*
2	Gga_rs14248546	125,490,179	BW42, BW63, ADBWG	*TRIQK*
2	Gga_rs15168561	136,710,388	BW28, BW42, BW63, ADBWG	*ENPP2*, *TAF2*, *DSCC1*, ***DEPTOR***, *COL14A1*
2	Gga_rs16088599	103,517,528	BW42, BW63, ADBWG	*OSBPL1A*, *IMPACT*, *ZNF521*
3	Gga_rs14356736	48,921,434	BW63, ADBWG, DCW, BrW	*PLEKHG1*, *MTHFD1L*, *AKAP12*, *ZBTB2*, *RMND1*, *ARMT1*, *CCDC170*, *ESR1*
4	Gga_rs13516467	38,746,248	BW63, ADBWG, DCW, BrW, DW	*NPNT*, *GSTCD*, *INTS12*, *ARHGEF38*, ***PPA2***, *TET2*
4	GGaluGA246480	12,518,793	DCW, BrW, DW	*SLC16A2*, *RLIM*, *NEXMIF*, *gga-mir-1573*, *ABCB7*, *UPRT*, *ZDHHC15*
7	Gga_rs13737657	14,269,161	BW42, BW63, ADBWG, DCW, BrW, DW	*U4*, *PDE1A*, *PPP1R1C*, *ITPRID2*, *NEUROD1*, *ITGA4*
7	Gga_rs14622272	28,057,143	BW42, BW63, ADBWG	*KALRN*, *ACADL*, *UMPS*, *ITGB5*, *HEG1*, *MYL1*, *ZNF148*, *SNX4*, *OSBPL11*, *LMLN*, *DTX3L*
7	Gga_rs14622611	28,327,789	BW42, BW63, ADBWG	*MYL1*, *OSBPL11*, *LMLN*, *DTX3L*, *PARP9*, *LANCL1*, *FAIM*, *CEP70*, *ESYT3*, *CFAP221*, *SCTR*, *TMEM37*, *DBI*, *C7H2ORF76*, *STEAP3*, *CPS1*, *C1QL2*, *MARCO*, *EN1*
7	Gga_rs15848860	14,393,379	BW42, BW63, ADBWG, DCW, BrW, DW	*U4*, *PDE1A*, *PPP1R1C*, *ITPRID2*, *NEUROD1*, *ITGA4*
7	GGaluGA308586	2,639,082	BW42, BW63, ADBWG, DCW, DW	*CNTNAP5*, *MAP2*, *MRAS*, *gga-mir-3530*, *TMEM177*, *PTPN4*, *EPB41L5*, *RALB*, *INHBB*, *GLI2*, ***UNC80***, *TFCP2L1*, *CLASP1*, *NIFK*, *TSN*, *IQCB1*, *EAF2*, *SLC15A2*, *HSPBAP1*, *SLC49A4*, *SEMA5B*, *PDIA5*, *SEC22A*, *ADCY6*, *KANSL1L*, *HACD2*, *MYLK*, *CCDC14*, *KALRN*, *ACADL*, *UMPS*, *ITGB5*, *HEG1*, *MYL1*, *ZNF148*, *SNX4*, *OSBPL11*, *LMLN*, *DTX3L*, *PARP9*, *LANCL1*, *FAIM*, *CEP70*, *ESYT3*, *CFAP221*, *SCTR*, *TMEM37*, *DBI*, *C7H2ORF76*
8	Gga_rs16640785	22,847,287	BW63, ADBWG, DCW, BrW	*TRABD2B*, *SLC5A9*, *SPATA6*, *gga-mir-1809*
8	GGaluGA330152	22,760,396	BW42, BW63, ADBWG	*TRABD2B*
9	Gga_rs15947559	11,450,206	DCW, BrW, DW	*PLOD2*
13	Gga_rs15677377	8,879,549	BW63, ADBWG, DCW, DW	*TTC1*, *ADRA1B*, *IL12B*, *FBXO38*, *HTR4*, *gga-mir-458a*, *SLC26A2*
13	Gga_rs15679261	8,271,910	BW42, BW63, ADBWG	*GABRB2*, *ATP10B*
13	Gga_rs15680269	7,909,523	BW42, BW63, ADBWG	-
13	GGaluGA093626	9,139,110	BW63, ADBWG, DCW	*gga-mir-458a*, *HTR4*, *SLC26A2*, *CSNK1A1*, *gga-mir-145*, *gga-mir-143*, *IL17B*, *PCYOX1L*, *GRPEL2*, *AFAP1L1*, *ABLIM3*
14	Gga_rs15003767	2,062,529	BW42, BW63, ADBWG	*FAM20C*, *FOXL3*
15	GGaluGA109523	8,381,798	BW63, ADBWG, DCW, BrW, DW	*DGCR2*, *VPS29L*, *VPREB3*, *CHCHD10*, *MMP11*, *SMARCB1*, *DERL3*, *SLC2A11*, *SLC2A11L1*, *MIF*, ***DDX51***, *GSTT1*, *DDTL*, *CABIN1*, *TBX6*, *CRKL*
17	Gga_rs14102454	3,408,140	BW63, ADBWG, DCW, DW	***PAPPA***, *ASTN2*
18	Gga_rs16347495	9,967,210	DCW, BrW, DW	*TIMP2*, *USP36*, *CYTH1*, *PGS1*, *SOCS3*, *AFMID*, *TK1*, *SYNGR2*, *TMC6*, *ARL16*, *HGS*, *MRPL12*, *GCGR*, *MCRIP1*, *PPP1R27*, *P4HB*, *ARHGDIA*, *ALYREF*, *NPB*, *PCYT2*, *SIRT7*, *MAFG*, *PYCR1*, *NME1*, *SPAG9*, *PITPNM3*, *FBXO39*, *TEKT1*, *SMTNL2*
19	GGaluGA126188	4,370,123	DCW, BrW, DW	*CUX1*, *PRKRIP1*, *ORAI3*, *ALKBH4*, *LRWD1*, *RASA4B*, *UPK3B*, *DTX2*, ***SSC4D***, *YWHAG*, *HSPB1*, *SRRM3*, *MDH2*, *TMEM120A*, *POR*, *TAF15*, *MMP28*, *RASL10B*, *AP2B1*
21	Gga_rs15182225	2,760,476	BW42, BW63, ADBWG	*TNFRSF18*, *gga-mir-429*, *gga-mir-200a*, *gga-mir-200b*, *gga-mir-6680*, *C1orf159*
23	GGaluGA188509	2,994,311	BW42, BW63, ADBWG, DCW, BrW, DW	*EPB41*, *TMEM200B*, *SRSF4*, *MECR*, ***PTPRU***, *gga-mir-1724*, *PTPRU*
27	Gga_rs13620324	4,812,782	BW63, ADBWG, BrW	*CRHR1*, *ITGB3*, *METTL2B*, ***TLK2***, *MRC2*, *TANC2*
28	Gga_rs14306444	1,714,462	BW42, BW63, ADBWG	*ZBTB7A*, *PIAS4*, *EEF2*, *gga-mir-1434*, *NMRK2*, *ATCAY*, *NRTN*, *DUS3L*, *LARP6L*, *RFX2*, *ACSBG2*, *MLLT1*, *ACER1*, *ANP32B*, *ZNF414*, *MYO1F*, *ADAMTS10*, *gga-mir-6615*, *ZAP70*
28	Gga_rs14306581	1,592,968	BW42, BW63, ADBWG	*NCLN*, *CELF5*, *HSD11B1L*, *MICOS13*, *gga-mir-1774*, *FSD1*, *YJU2*, *gga-mir-6593*, *ZBTB7A*, *PIAS4*, *EEF2*, *gga-mir-1434*, *NMRK2*, *ATCAY*, *NRTN*, *DUS3L*, *LARP6L*, *RFX2*, *ACSBG2*, *MLLT1*

^1^ GGA, *Gallus gallus* chromosomes. ^2^ Traits: BW42, body weight (BW) at 42 days; BW63, BW at 63 days; ADBWG, average daily body weight gain; DCW, dressed carcass weight; BrW, breast weight; DW, drumstick weight. Prioritized candidate genes localized at the positions of SNPs identified for three and more traits are highlighted in bold.

## Data Availability

The genotyping data presented in this study can be shared with the third parties upon approval with the GWMAS Consortium. Other original contributions presented in the study are included in the article and Supplementary Materials; further inquiries can be directed to the corresponding authors with the permission provided by the chickens’ owners.

## Supplementary Materials

The following supporting information can be downloaded at: https://www.mdpi.com/article/10.3390/genes15101246/s1, Table S1: List of SNPs associated with growth and meat productivity indicators in F_2_ roosters of the resource population; Table S2: Gene ontology (GO) term enrichment analysis at the positions of the determined SNPs in F_2_ roosters of the resource population.
